# A narrative review of *in vitro* studies on the role of reactive oxygen species in aggravating high-grade serous ovarian cancer development

**DOI:** 10.3389/fonc.2026.1878450

**Published:** 2026-07-31

**Authors:** Jeongmin Lee, Seung Geun Yeo, Hye Ok Kim, Jae Min Lee, Manish Kumar Singh, Sung Soo Kim, Tong In Oh, Dong Choon Park

**Affiliations:** 1 Department of Medicine, College of Medicine, Kyung Hee University, Seoul, Republic of Korea; 2 Clinical Research Institute, Kyung Hee University Medical Center, Seoul, Republic of Korea; 3 Department of Otorhinolaryngology – Head and Neck Surgery, College of Medicine, Kyung Hee University Medical Center, Kyung Hee University, Seoul, Republic of Korea; 4 Department of Biochemistry and Molecular Biology, College of Medicine, Kyung Hee University, Seoul, Republic of Korea; 5 Department of Biomedical Engineering, College of Medicine, Kyung Hee University, Seoul, Republic of Korea; 6 Department of Obstetrics and Gynecology, St. Vincent’s Hospital, College of Medicine, The Catholic University of Korea, Seoul, Republic of Korea

**Keywords:** chemoresistance, epithelial-mesenchymal transition, ovarian cancer, oxidative stress, reactive oxygen species

## Abstract

Ovarian cancer is often diagnosed at an advanced stage because its early symptoms are nonspecific, and the prognosis remains poor due to frequent recurrence and the development of drug resistance even after standard treatment. This review includes 19 *in vitro* studies and seeks to mechanistically summarize how reactive oxygen species (ROS) reinforce the tumorigenic program from the early stages of ovarian carcinogenesis to tumor progression, metastasis, and treatment resistance. Collectively, the selected studies suggest that exposure to environmental toxicants can increase ROS levels in ovarian epithelial cells, accompanied by inflammatory responses and elevated DNA damage markers, thereby creating a carcinogenesis-priming environment. During the progression stage, hypoxia, hormones, growth factors, and lipid signaling repeatedly activate survival and growth pathways, such as those involving HIF-1α/VEGF, JAK/STAT3, and AKT/mTOR, through ROS-mediated mechanisms to promote tumor cell proliferation, anti-apoptotic activity, and angiogenesis. In addition, ROS are associated with alterations in epithelial-to-mesenchymal transition-related markers, remodeling of the extracellular matrix, and regulation of matrix metalloproteinases, which enhance the metastatic and invasive potential of tumor cells. In the therapeutic context, ROS have been suggested to contribute to platinum-based chemotherapy resistance through mechanisms including changes in mitochondrial dynamics, activation of the DNA damage response, and reprogramming of ROS-dependent phosphorylation networks. Understanding the diverse molecular mechanisms and clinical manifestations associated with ROS expression in high-grade serous ovarian cancer (HGSOC) will contribute to a more precise understanding of its pathophysiology. Furthermore, studies on redox-targeted therapeutic strategies, particularly the application of mitochondria-targeted antioxidants, may provide valuable translational insights for the treatment of ovarian cancer and other ROS-related diseases.

## 1 Introduction

Ovarian cancer is one of the most lethal gynecologic malignancies, but its diagnosis is often delayed because early symptoms are nonspecific in many patients. In clinical practice, ovarian cancer is frequently detected at an advanced stage, often accompanied by peritoneal dissemination or extensive intra-abdominal lesions at the time of diagnosis, complicating curative treatment. The standard treatment strategy centers on maximal cytoreductive surgery followed by platinum-based chemotherapy. However, recurrence and drug resistance frequently occur even after treatment, representing major limitations to long-term survival. These clinical characteristics suggest that ovarian cancer should not be understood solely as the result of accumulated genetic mutations; it should also be interpreted in the context of tumor cells adapting to microenvironmental conditions such as hypoxia, inflammatory stimuli, hormonal and growth factor signaling, and metabolic stress acting together to reinforce survival and progression programs (1).

Reactive oxygen species (ROS) are a group of oxidative molecules generated through processes such as electron leakage from the mitochondrial electron transport chain and the activity of NADPH oxidase family enzymes. These reactive species include superoxide, hydrogen peroxide, and hydroxyl radicals. Because ROS can mediate signal transduction through mechanisms such as oxidation of cysteine residues in proteins, modulation of phosphorylation networks, and regulation of transcription factor activity, they are increasingly recognized not merely as metabolic byproducts but also as redox-sensitive signaling mediators that coordinate cellular responses (2). In recent years, the paradigm of ROS in cancer biology has rapidly evolved. Comprehensive updates in 2024 and 2025 emphasize that ROS not only govern baseline cellular homeostasis but also actively drive ovarian cancer progression, serving as emerging biomarkers for early diagnosis and prognosis, as well as crucial targets for novel therapeutic innovations (3, 4). In cancer, increased ROS production and the reorganization of antioxidant defense systems occur simultaneously, and tumor cells are thought to maintain a growth- and survival-favorable state by regulating this redox balance (5).

In high-grade serous ovarian cancer (HGSOC), the axis of oxidative stress and ROS has been discussed as a pathophysiological mechanism across multiple stages, including tumor initiation, progression, metastasis, and therapeutic resistance. At the initiation stage, ROS can increase oxidative damage to DNA, proteins, and lipids to promote genomic instability and, when combined with inflammatory signaling, contribute to forming a carcinogenic environment. During tumor progression, ROS may interact with hypoxic responses, leading to stabilization of HIF-1α and increased expression of VEGF, which ultimately promotes angiogenesis and adaptive tumor survival (6). Furthermore, emerging evidence highlights that ROS directly mediate ovarian cancer development, angiogenesis, and platinum resistance by activating specific downstream networks, such as the CXCL8 and GSK-3β/p70S6K1 signaling axes within the tumor microenvironment (7). In addition, ROS-generating enzymes such as NOX4 are reportedly associated with specific signaling axes linked to tumor growth and therapeutic resistance in ovarian cancer, suggesting that ROS may reinforce tumorigenic programs through defined molecular pathways rather than simply reflecting elevated oxidative stress (8). From the perspective of treatment response, ROS are also closely associated with the cytotoxic effects of platinum-based drugs. *In vitro* evidence indicates that cisplatin, which is well known to induce DNA damage, can also trigger mitochondrial dysfunction, leading to increased mitochondrial-derived ROS and downstream oxidative signaling that may participate in determining cell fate, including apoptosis (9). These findings indicate that treatment response and resistance development in ovarian cancer may be closely linked to alterations in redox status and remodeling of mitochondrial function. Because the biological effects of ROS vary depending on their chemical species, sources, intracellular localization, and temporal intensity, it has repeatedly been pointed out that their role in cancer cannot be reduced to a single directional effect (5). Within the pathophysiological context of HGSOC, accumulating evidence indicates that, under certain conditions, ROS can activate progression-related signaling pathways such as the HIF-1α/VEGF, STAT3, AKT/mTOR, and DNA damage response pathways. These pathways ultimately reinforce tumorigenic phenotypes, including proliferation, survival, angiogenesis, invasion, and therapeutic resistance (1, 2, 6). Therefore, as a follow-up to previous *in vivo* studies on ROS in HGSOC, this review selectively focuses on *in vitro* studies examining the mechanisms by which ROS reinforce stage-specific tumorigenic programs in HGSOC. In particular, this narrative review aims to explore how diverse upstream stimuli converge on common progression-related signaling circuits through ROS-mediated mechanisms.

## 2 Methods

Although numerous studies have investigated the role of ROS in cancer biology, the literature lacks a comprehensive structured review focusing specifically on *in vitro* experimental evidence supporting the pro-tumorigenic (tumor-promoting) roles of ROS in ovarian cancer. Therefore, a systematic search was conducted by one reviewer (J.L.) to identify relevant literature. Five electronic databases-PubMed, SCOPUS, Cochrane Library, EMBASE, and Google Scholar were searched for studies published between January 1990 and May 2025. The year 1990 was selected as the starting point because preliminary searches revealed that *in vitro* studies meeting our inclusion criteria primarily emerged from this period onward. This timeline also aligns with major methodological advancements in redox biology; prior to the 1990s, *in vitro* ROS assays generally lacked the specificity and sensitivity required to elucidate distinct intracellular tumorigenic mechanisms. The subsequent standardization of key technologies, such as advanced fluorescent probes (e.g., DCFH-DA variants) and specific ROS scavengers, enabled the precise experimental validations reviewed herein. The search strategy used combinations of the following keywords: “ovarian cancer”, “reactive oxygen species”, and “ROS”, along with Boolean operators (“AND”, “OR”) to refine results.

The search was limited to English-language publications. The inclusion criteria were:

1. Original research articles,
2. Studies investigating ROS or free radical-related mechanisms in ovarian cancer,
3. Studies that included *in vitro* experiments, whether alone or in combination with *in vivo* data, and
4. Studies in which ROS (or ROS-generating/redox-regulatory pathways) were experimentally linked to pro-tumorigenic outcomes, including enhanced proliferation/survival, angiogenic signaling, EMT/migration/invasion, or therapeutic resistance, and/or in which ROS suppression (e.g., antioxidants, inhibition of ROS sources, or genetic perturbation of redox regulators) attenuated these malignant phenotypes.

The exclusion criteria were as follows:

1. Off-topic studies unrelated to HGSOC.
2. Studies lacking *in vitro* data.
3. Review articles.
4. Studies that did not directly address ROS or redox signaling.
5. Duplicate publications.
6. *In vitro* studies presenting ROS induction or elevation primarily as anti-tumorigenic (e.g., promoting apoptosis, inhibiting growth, or increasing therapeutic sensitivity). These anti-tumorigenic studies were excluded because they fell outside the predefined scope of this review. This specific criterion was applied to maintain a strict focus on synthesizing pro-tumorigenic mechanisms, rather than to negate the context-dependent, dual roles of ROS in cancer biology.

**Figure 1:**
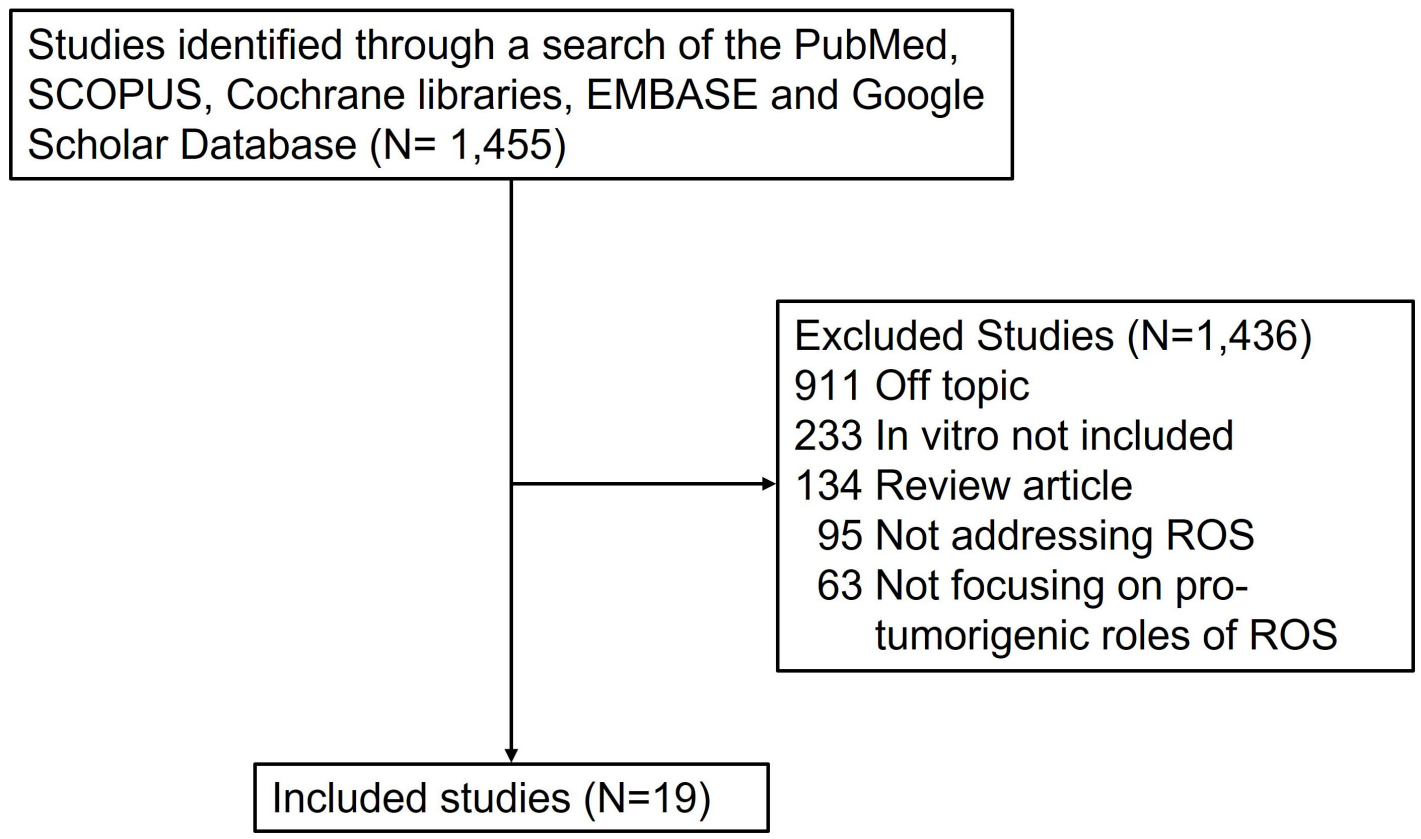
Diagram of the literature-search workflow.

From an initial pool of 1,455 studies, a total of 1,436 were excluded based on the established criteria: 911 for being off-topic, 233 for lacking *in vitro* experiments, 134 for being review articles, 95 for not addressing ROS, and 63 for not focusing on the pro-tumorigenic roles of ROS. Consequently, 19 studies were ultimately included for analysis in this review (**Figure 1**).

## 3 Discussion

ROS are chemically reactive molecules that perform essential functions in biological systems (10), but act as a double-edged sword. Moderate increases in ROS can promote cell proliferation and differentiation (11), whereas excessive ROS can cause oxidative damage to lipids, proteins, and DNA (12). Therefore, maintaining ROS homeostasis is critical for normal cellular growth and survival. In normal cells, ROS levels are regulated through a balance between ROS-generation and -removal systems, the latter of which include antioxidant enzymes such as superoxide dismutase, glutathione peroxidase, peroxiredoxin, glutaredoxin, thioredoxin, and catalase.

ROS levels are generally elevated in cancer cells, and this increase is considered to play an important role in tumor initiation and progression (13). Disruption of redox homeostasis in cancer cells results from increased ROS production or reduced ROS-scavenging capacity, and yields a condition commonly referred to as oxidative stress (10). Cancer cells experiencing elevated oxidative stress may be more vulnerable to additional ROS-induced damage triggered by external factors. Accordingly, modulation of ROS levels through redox regulation has been proposed as a strategy to selectively induce cancer cell death without causing severe toxicity to normal cells.

Numerous studies have reported altered levels of ROS-scavenging enzymes, such as superoxide dismutase (SOD), glutathione peroxidase, and peroxiredoxin, in malignant cells and primary tumor tissues. Antioxidant enzymes are overexpressed in the majority (approximately 70–80%) of ovarian and endometrial cancer tissues, and their expression levels are positively correlated with disease progression. These findings suggest that redox homeostasis and stress adaptation are abnormally regulated in cancer cells. Taken together, the results reported to date indicate that both ROS and antioxidant enzymes are increased in cancer cells, and that the enhanced antioxidant capacity of cancer cells represents an adaptive response to intrinsic oxidative stress, which may contribute to drug resistance (14). Therefore, targeting oxidative stress may have important therapeutic implications (**Table 1**).

**Table 1:** *In vitro* studies on reactive oxygen species in HGSOC.

Author/ year/ Reference	Sample	Detection method	Target substance(s) associated with ROS	Results/Conclusions
Shah et al., 2020 (15)	HOSE (human ovarian surface epithelial) cells (Normal)	H_2_DCFDA fluorescence assay (intracellular ROS), MTT assay, qPCR, ELISA, Comet assay	Intracellular ROS (unspecified), IL-6, IL-1β, TNF-α, NF-κB, COX-2	β-HCH, DDE, and Dieldrin (20 μM) significantly increased ROS generation (↑65–81%, p < 0.05–0.01). ROS increase was associated with upregulation of inflammatory mediators (e.g., IL-6 ↑7.6–10.8-fold; NF-κB ↑6.4–11.7-fold) and DNA damage (comet assay, p < 0.01). /OCPs induce oxidative stress, inflammation, and DNA damage in HOSE cells, suggesting a role in ovarian cancer initiation.
Cohen et al., 2016 (16)	Primary human SOC tissue samples: Normal (Normal), Cystadenoma/Borderline (Benign/Precancerous), Malignant stage III/IV (HGSOC)	DCFH-DiOxyQ probe, MDA assay, DNPH assay, Western blot (GPX3)	ROS, MDA, Reactive carbonyl proteins, GPX3	ROS levels were 96% higher in malignant tissues compared to normal tissues (p=0.007). MDA levels were 5-fold higher in malignant vs. non-diseased tissues (p=0.019). Reactive carbonyl proteins were significantly elevated (p=0.009) and the GPX3 antioxidant enzyme was decreased in malignant tissues. ROS levels were ~9% higher in African-American vs. Caucasian patients. /ROS accumulation and oxidative damage correlate with tumor progression. Antioxidant depletion (GPX3) may contribute to malignancy development in SOC.
Nguyen & Syed, 2011 (17)	OVCA420, OVCA429 (HGSOC); ECC-1, Ishikawa (Endometrial cancer)	Aminophenyl fluorescein (APF) for intracellular ROS, Western blot (SOD1, SOD2, GPx1, catalase, Trx1), Oxidative stress markers (protein carbonyl, MDA, nitrotyrosine)	Intracellular ROS (measured by APF), SOD1, GPx1, catalase, Trx1, protein carbonyl, MDA, nitrotyrosine, p53, BAX, BCL-2	Cancer cells exhibited higher basal ROS than normal cells; P4 treatment time-dependently reduced ROS manner (significant after 6–24 h, p<0.05). P4 decreased oxidative stress markers (MDA, protein carbonyl, nitrotyrosine) and antioxidant enzyme levels (SOD1, catalase, GPx1, Trx1). Apoptosis was induced via ↑p53, ↑BAX, ↓BCL-2, alongside caspase-3 activation. All effects were reversed by a progesterone receptor antagonist (mifepristone). /Progesterone suppresses ROS generation and oxidative stress, leading to apoptosis via the p53–BAX–BCL-2 axis in ovarian and endometrial cancer cells.
Maleki et al., 2015 (28)	OVCAR-3 human ovarian cancer cells (HGSOC)	DCFH-DA (total intracellular ROS), Griess method (NO), MTT assay	Reactive oxygen species (peroxides, via DCF), nitric oxide (NO)	17β-Estradiol (E2) treatment (100 nM) significantly and dose-dependently increased intracellular ROS (p < 0.001) and NO levels in OVCAR-3 cells. ROS production was inhibited by the antioxidants, NAC and Ebselen, and the estrogen receptor antagonist ICI182780 (p < 0.001). Co-treatment with progesterone also reduced ROS and NO generation (p < 0.001 vs. E2 alone). Increased ROS levels paralleled the increased cell viability, suggesting that ROS is involved in E2-induced proliferative signaling. /E2 promotes ovarian cancer cell proliferation via ROS and NO production, likely through estrogen receptor-mediated pathways. ROS may function as a mitogenic signal in hormone-responsive tumors.
Chen et al., 2024 (29)	A2780, SKOV3 (Non-HGSOC); MIHA (Liver); IOSE-80 (Normal ovary)	H_2_DCFDA assay, Western blot, siRNA knockdown, MTT assay, JC-1, Flow cytometry	HIF-1α, ROS (H_2_O_2_), JAK/STAT3, BCL2, MMP9	HIF-1α induced intracellular ROS (mainly H_2_O_2_, p<0.01) and activated JAK/STAT3 signaling in ovarian cancer cells. ROS mediated HIF-1α-induced proliferation, invasion, and anti-apoptosis. NAC suppressed ROS and reversed these effects. /HIF-1α promotes ovarian cancer progression by increasing intracellular H_2_O_2_ to activate JAK/STAT3 signaling and enhance tumor cell survival and invasiveness. ROS acts as a key mediator in this oncogenic pathway.
Zhang et al., 2013 (30)	ES2, Hey human ovarian epithelial cancer cells (Non-HGSOC)	DCFH-DA (ROS detection), Western blot (Nrf2, HIF1α), ELISA (VEGF), ChIP assay (HIF1α–VEGF promoter binding), siRNA knockdown (Nrf2, HIF1α)	ROS (including H_2_O_2_), Nrf2, HIF1α, VEGF, FSH, NAC	FSH significantly and time- and dose-dependently increased intracellular ROS and upregulated Nrf2 and HIF1α expression (p < 0.05). The ROS scavenger, NAC, blocked FSH-induced Nrf2 activation, HIF-1α activation, and VEGF expression (p < 0.05). Knockdown of Nrf2 or HIF-1α reduced VEGF levels, and their dual knockdown led to a more significant inhibition. FSH enhanced HIF-1α binding to the VEGF promoter, and this was blocked by NAC, siNrf2, or siHIF-1α. /FSH stimulates VEGF expression in ovarian cancer cells through a ROS–Nrf2–HIF1α axis. ROS, particularly H_2_O_2_, act as upstream mediators to trigger Nrf2 activation and subsequent HIF1α-driven VEGF transcription. Disruption of this axis may provide a therapeutic approach in ovarian cancer.
Liu et al., 2006 (31)	OVCAR-3 (HGSOC)	DCFH-DA assay (H_2_O_2_), RT-PCR, Western blot, Luciferase assay, Transfection (HIF-1α, p70S6K1)	Hydrogen peroxide (H_2_O_2_), AKT, p70S6K1, VEGF, HIF-1α	EGF (40 ng/mL) treatment increased H_2_O_2_ production in OVCAR-3 cells (P<0.05), and this was abolished by catalase. EGF-induced AKT and p70S6K1 phosphorylation was ROS-dependently inhibited by catalase (P<0.05). Catalase dose-dependently suppressed VEGF mRNA expression and VEGF promoter activation (P<0.05 vs. EGF-treated). EGF increased HIF-1α but not HIF-1β; this was reversed by catalase and rapamycin (P<0.05). Forced expression of HIF-1α or p70S6K1 reversed catalase- or rapamycin-mediated inhibition of VEGF transcription. /EGF stimulates angiogenic gene expression (VEGF, HIF-1α) in ovarian cancer cells via H_2_O_2_-mediated activation of AKT/p70S6K1. H_2_O_2_ acts as a key second messenger.
Xia et al., 2007 (32)	OVCAR-3 (HGSOC); A2780 (Non-HGSOC); IOSE 386/397 (Normal ovarian epithelial cells)	CM2-DCFHDA (DCFH-DA) fluorescence assay (intracellular H_2_O_2_), Western blot (HIF-1α, VEGF), RT-PCR (NOX4, VEGF), Luciferase assay (VEGF promoter activity), siRNA (p47^phox, NOX4)	H_2_O_2_, NOX4, p47^phox, HIF-1α, VEGF, DPI, rotenone, catalase, GPx	Ovarian cancer cells showed 5–6x higher ROS than normal epithelial cells (p < 0.01). ROS were reduced by DPI, rotenone, and siRNA against NOX4 or p47^phox. Catalase (an H_2_O_2_ scavenger) inhibited HIF-1α and VEGF expression, whereas H_2_O_2_ treatment increased HIF-1α levels. Luciferase assays confirmed that VEGF transcription was HIF-1α and ROS dependent. (p < 0.05 in all inhibitor treatments). /Endogenous ROS, particularly H_2_O_2_ generated via NOX4, regulate VEGF expression and HIF-1α stability in ovarian cancer cells. ROS signaling is essential for tumor-associated angiogenesis and growth, implicating redox pathways as therapeutic targets.
Saunders et al., 2011 (33)	SKOV3 human ovarian cancer (Non-HGSOC)	DCF-DA fluorescence (total ROS), Western blot (p-Akt, p-ERK), SEAP NF-κB reporter assay	H_2_O_2_ (explicitly stated), NADPH oxidase (NOX), p-Akt, p-ERK, NF-κB	LPA (10 nM) significantly increased ROS generation (via DCF-DA) in SKOV3 cells (p < 0.007). This ROS increase was blocked by NOX inhibitors DPI, apocynin, and the scavengers, PEG-catalase and EUK-134, implicating NADPH oxidase and H_2_O_2_ as being central to LPA signaling. ROS generation preceded and mediated Akt and ERK phosphorylation, both of which were suppressed by antioxidant treatments (p < 0.01). NF-κB transcriptional activity was upregulated by LPA and inhibited by antioxidants (NAC, curcumin, EUK-134), with strong statistical significance (p < 0.003 to < 0.008). /H_2_O_2_ generated by NOX is essential for LPA-induced survival and proliferative signaling via Akt, ERK, and NF-κB pathways in SKOV3 ovarian cancer cells.
Zhao et al., 2021 (34)	Human ovarian cancer cell lines: OVCAR3 (HGSOC), HEY (Non-HGSOC)	qRT-PCR, Western blot, BODIPY 493/503 staining, flow cytometry (ROS), MitoTracker, MTS assay, colony formation, wound healing, transwell invasion, inhibitor/activator treatment (NAC, H_2_O_2_, MK2206, SC79)	ROS, p-AKT, p-mTOR, SREBP1/2, ACC1, FASN, SCD1, HMGCS1, HMGCR	Overexpression of MIEF2 in HEY cells increased mitochondrial ROS (↑DCF fluorescence), activated AKT/mTOR (↑p-AKT, ↑p-mTOR), and upregulated lipogenic genes (SREBP1/2, ACC1, FASN, SCD1, HMGCS1, HMGCR). This led to increases in intracellular fatty acids, triglycerides, cholesterol, and phospholipids (all p < 0.01). NAC reversed the effect on ROS and signaling activation. Inhibitors (MK2206, rapamycin) blocked MIEF2-induced SREBP1/2 activation and lipid accumulation. /MIEF2 promotes the ROS-dependent activation of AKT/mTOR signaling, leading to SREBP1/2-mediated lipogenic reprogramming in ovarian cancer cells. ROS acts as a crucial upstream effector in this pathway. The findings suggest that MIEF2 is a potential therapeutic target for interrupting ROS-driven oncogenic lipid metabolism in ovarian cancer.
Hu et al., 2005 (35)	SKOV3 ovarian cancer cells (Non-HGSOC)	Western blot, Flow cytometry (HE probe for O_2_^-^), Colony formation assay	Mn-SOD (SOD2), Superoxide (O_2_^-^), ROS	Mn-SOD knockdown via siRNA resulted in a 70% increase in intracellular superoxide (O_2_^-^) (measured by HE probe). Increased O_2_^-^ led to enhanced cell proliferation in a colony formation assay (p < 0.01). Cu,Zn-SOD (SOD1) levels remained unchanged, that this effect was specific for Mn-SOD. /Mn-SOD suppresses cell proliferation in ovarian cancer cells by scavenging superoxide. Its suppression significantly and ROS-dependently increases intracellular O_2_^-^ and enhances growth.
Worley et al., 2019 (36)	OVCAR3 human ovarian cancer cells (HGSOC)	Amplex Red assay (extracellular HO), Clonogenicity assay, Crystal violet viability assay, Ultra-low attachment (ULA) spheroid assay, Live/dead staining	GPx3, extracellular H_2_O_2_, ascorbate	GPx3 knockdown significantly reduced clonogenicity and survival under anchorage-independent conditions (p < 0.01–0.0001). GPx3-KD cells were more sensitive to ascorbate-induced cytotoxicity (IC_50_: 0.22–0.26 mM vs. 0.42 mM in control; p < 0.01). Amplex Red assays showed significantly higher extracellular H_2_O_2_ in GPx3-KD cells after ascorbate treatment (p < 0.0001). The survival advantage of GPx3-KD cells was lost in patient ascites regardless of sORP or Fe²^+^ levels (p < 0.001). /GPx3 is critical for scavenging extracellular H_2_O_2_ and enabling ovarian cancer cell survival under oxidative stress, particularly in the ascites environment. High GPx3 expression may support transcoelomic metastasis by protecting cells from exogenous ROS.
Karlsson et al., 2024 (37)	OVCAR-3, OVCAR-8 human ovarian cancer cells (HGSOC)	DCFH-DA (ROS), Western blot, Flow cytometry, RNA-seq	ROS, SOD1, Thioredoxin (TXN),	CRISPR/Cas9-mediated SOD1 knockout significantly increased ROS levels in OVCAR-3 and OVCAR-8 cells (↑ MFI, p < 0.001), accompanied by upregulation of compensatory antioxidant genes (TXN, PRDX6, GPX1). Despite this compensation, SOD1-KO cells showed reduced proliferation and increased apoptosis (sub-G1 population ↑ 2.5-fold, p < 0.01) compared to the control. /SOD1 is essential for redox homeostasis in ovarian cancer. Its loss triggers ROS accumulation and cell death despite antioxidant gene upregulation, confirming the functional importance of SOD1 in ROS defense.
Wang et al., 2014 (38)	SKOV3 human ovarian cancer cells (Non-HGSOC)	DCFH-DA fluorescence assay (intracellular ROS) Western blot (HIF-1α, LOX, E-cadherin) RT-PCR siRNA transfection (HIF-1α) Wound healing assay	Emodin (ROS inducer), DTT (ROS scavenger), HIF-1α, LOX, E-cadherin, β-APN (LOX inhibitor)	Emodin treatment significantly increased intracellular ROS levels (+55.7%, p < 0.01) and upregulated HIF-1α and LOX expression while downregulating E-cadherin. These effects were reversed by DTT, HIF-1α knockdown, or LOX inhibition (p < 0.05). Emodin also enhanced cell migration, which was inhibited by the same interventions (p < 0.01). /ROS function as upstream activators of the HIF-1α/LOX/E-cadherin axis, promoting epithelial-mesenchymal transition (EMT) and migration in ovarian cancer cells. Targeting ROS may disrupt this pro-metastatic signaling cascade.
Zhou et al., 2017 (39)	Human ovarian cancer cell line: SKOV3-PM4 (Non-HGSOC, highly lymphatic metastatic)	MTT assay, Wound healing, Transwell invasion, Western blot, DCFH-DA (ROS), Nitroblue tetrazolium assay (NADPH oxidase), p38/JNK/AP-1, Rac1 activator (PMA), Rac1 inhibitor (NSC23766)	ROS, NADPH oxidase, Rac1, MMP-2/3/7/9/19, TIMP-1/2, NM23-H1, JNK, p38, AP-1	Rhein (7.7–11.3 µM) significantly inhibited the proliferation, migration, and invasion of SKOV3-PM4 cells (p < 0.01). PMA increased NADPH oxidase activity and ROS production, but this effect was reversed by rhein treatment. Rhein reduced MMP-2, -3, -7, -9, -19 and phosphorylated JNK and AP-1, while upregulating TIMP-1/2 and NM23-H1. The effects of rhein were similar to those of NSC23766-induced Rac1 inhibition. /Rhein inhibits ovarian cancer cell migration and invasion through suppression of Rac1-mediated ROS generation and downstream MAPK (JNK/p38)/AP-1 signaling. This leads to decreased MMP levels and increased metastasis-suppressing protein levels, indicating that targeting the Rac1/ROS/MAPK axis may be an effective strategy against metastatic ovarian cancer.
Han et al., 2019 (40)	Human ovarian cancer cell lines: SKOV3, PA1 (Non-HGSOC); OVCAR3 (HGSOC); Patient-derived spheroids (PDS)	MTT assay, Annexin V/PI, DCFH-DA (H_2_O_2_), DHE (O_2_^-^), Western blot (Drp1, Mfn1, PARP), Immunofluorescence, TUNEL assay	Hypoxia-induced ROS (H_2_O_2_), Drp1, p-Drp1 (Ser616/637), Mfn1	Under hypoxia, intracellular H_2_O_2_ increased, promoting mitochondrial fission via ↓ p-Drp1 (Ser637) and ↓ Mfn1. This shift induced cisplatin (CDDP) resistance. Exogenous H_2_O_2_ mimicked the effect, while ROS scavengers (NAC, Trolox) reversed mitochondrial fission and restored sensitivity. Inhibition of fission via Mdivi-1 or si-Drp1 increased apoptosis and restored CDDP sensitivity even under hypoxia. Patient-derived spheroids showed same pattern: In them, Mdivi-1 + CDDP significantly reduced tumor size and viability. /Hypoxia-induced ROS drives CDDP resistance via mitochondrial fission, and targeting Drp1 may restore chemosensitivity.
Meng et al., 2018 (41)	SKOV3, SKOV3-CR (Non-HGSOC); PEO14, PEO23 (HGSOC)	ROS imaging assay (not further specified), Western blot, qPCR, siRNA knockdown, Cell viability assays	ROS (unspecified), DUOXA1, ATR, pATR, Chk1, pChk1, γH2AX	DUOXA1 overexpression in SKOV3-CR and PEO23 cells led to significantly higher ROS levels compared to those in parental lines (p < 0.001). The ROS inhibitor, YCG063, decreased ROS and suppressed phosphorylation of ATR (Thr-1989), Chk1 (Ser-317), and γH2AX (p < 0.001). DUOXA1 knockdown reduced ROS and ATR-Chk1 activation and re-sensitized resistant cells to cisplatin (p < 0.05). ROS elevation sustained DNA damage response signaling (ATR-Chk1) and promoted G2/M checkpoint activation and resistance. /DUOXA1 promotes cisplatin resistance by elevating intracellular ROS, which sustains the ATR-Chk1-mediated DNA damage response in ovarian cancer cells.
Li et al., 2022 (42)	A2780cisR, SKOV3cisR cisplatin-resistant ovarian cancer cells (Non-HGSOC)	ROS: CM-H2DCFDA, H_2_O_2_/superoxide assay Enzyme activity: CAMK2G/ITPKB kinase assay, IP4 assay Cell viability: IC50 assay Gene/protein modulation: shRNA, overexpression, mutation (WT/MV, S174A/D)	H_2_O_2_, superoxide, CAMK2G, ITPKB (pS174), NOX4	Cisplatin-resistant cells exhibited increased mitochondrial ROS (H_2_O_2_, superoxide), which activated CAMK2G via oxidation (Met281/282). CAMK2G phosphorylated ITPKB at Ser174, leading to IP4 production and suppression of NOX4-derived ROS. CAMK2G inhibition or mutation impaired this axis and re-sensitized cells to cisplatin (p < 0.001). Phospho-deficient ITPKB (S174A) failed to suppress ROS or confer resistance. ROS modulators (NAC, H_2_O_2_) altered this signaling, confirming ROS-dependence. /ROS sensing via CAMK2G and downstream ITPKB phosphorylation modulate ROS homeostasis and cisplatin resistance in ovarian cancer.
Chan et al., 2008 (43)	HOSE (Normal); OVCAR3, OV420, OV429, OV433 (HGSOC); OV2008, C13*, A2780s, A2780cp, SKOV3, DOV13-5 (Non-HGSOC)	qRT-PCR, semi-qRT-PCR, Western blot (MKP3, p-ERK1/2), H2DCFDA intracellular H_2_O_2_ (plate reader), XTT viability assay, soft agar (anchorage-independent growth), shRNA/siRNA knockdown, MKP3 overexpression, *in vivo* ubiquitination assay, proteasome inhibitor (MG132), antioxidant (NAC), cisplatin (CDDP), MEK inhibitor (PD98059)	H_2_O_2_(oxidative stress), NAC (ROS scavenger), MG132 (proteasome inhibitor), cisplatin (CDDP), PD98059 (MEK1/2 inhibitor)	Higher intracellular H_2_O_2_ was found in cisplatin-resistant/MKP3-low cells: A2780cp and C13* cells showed significantly higher intracellular H_2_O_2_ than their cisplatin-sensitive counterparts (A2780cp: P = 0.01; C13*: p = 0.001). More tumorigenic phenotype in resistant cells: Cisplatin-resistant cells proliferated faster (A2780cp: p = 0.025; C13*: p = 0.005) and A2780cp cells formed more soft-agar colonies than A2780s cells (p < 0.001). MKP3 loss promotes tumorigenicity: shRNA knockdown of MKP3 in A2780s cells increased proliferation (p < 0.001) and anchorage-independent growth (p < 0.01). MKP3 restoration suppresses tumorigenicity: MKP3 overexpression in A2780cp cells reduced proliferation (p < 0.01) and soft-agar growth (p < 0.001). ROS drives MKP3 proteasomal degradation: Proteasome inhibition (MG132) restored MKP3 and the antioxidant NAC inhibited MKP3 ubiquitination/degradation, supporting a ROS-dependent proteasome mechanism. /ROS accumulation (H_2_O_2_) promotes ovarian cancer tumorigenicity and chemoresistance by inducing ubiquitin–proteasome-mediated degradation of MKP3, which leads to aberrant ERK1/2 activation.

ACC1, Acetyl-CoA Carboxylase 1; AKT, Protein Kinase B; AP-1, Activator Protein 1; APF, Aminophenyl Fluorescein; ATR, Ataxia Telangiectasia and Rad3-related Protein; BAX, BCL2 Associated X Protein; BCL-2, B-cell Lymphoma 2; CAMK2G, Calcium/Calmodulin-Dependent Protein Kinase II Gamma; CDDP, Cisplatin (cis-diamminedichloroplatinum(II)); ChIP, Chromatin Immunoprecipitation; Chk1, Checkpoint Kinase 1; COX-2, Cyclooxygenase-2; CRISPR/Cas9, Clustered Regularly Interspaced Short Palindromic Repeats/CRISPR Associated Protein 9; DCFH-DA/H2DCFDA, 2’,7’-Dichlorodihydrofluorescein Diacetate; DDE, Dichlorodiphenyldichloroethylene; DNPH, 2,4-Dinitrophenylhydrazine; DPI, Diphenyleneiodonium; Drp1, Dynamin-related Protein 1; DTT, Dithiothreitol; DUOXA1, Dual Oxidase Maturation Factor 1; E2, 17β-Estradiol; EGF, Epidermal Growth Factor; ELISA, Enzyme-Linked Immunosorbent Assay; EMT, Epithelial-Mesenchymal Transition; ERK, Extracellular Signal-Regulated Kinase; FASN, Fatty Acid Synthase; FSH, Follicle-Stimulating Hormone; GPx/GPX3, Glutathione Peroxidase; HIF-1α, Hypoxia-Inducible Factor 1-alpha; HMGCR, 3-Hydroxy-3-Methylglutaryl-CoA Reductase; HMGCS1, 3-Hydroxy-3-Methylglutaryl-CoA Synthase 1; HOSE, Human Ovarian Surface Epithelial; IL, Interleukin; IP4, Inositol Tetrakisphosphate; ITPKB, Inositol-Trisphosphate 3-Kinase B; JAK, Janus Kinase; JNK, c-Jun N-terminal Kinase; KD, Knockdown; KO, Knockout; LOX, Lysyl Oxidase; LPA, Lysophosphatidic Acid; MAPK, Mitogen-Activated Protein Kinase; MDA, Malondialdehyde; MEK, Mitogen-Activated Protein Kinase Kinase; MFI, Mean Fluorescence Intensity; Mfn1, Mitofusin-1; MIEF2, Mitochondrial Elongation Factor 2; MKP3, Mitogen-Activated Protein Kinase Phosphatase 3; MMP, Matrix Metalloproteinase; mTOR, Mammalian Target of Rapamycin; MTT, 3-(4,5-Dimethylthiazol-2-yl)-2,5-diphenyltetrazolium Bromide; NAC, N-acetylcysteine; NF-κB, Nuclear Factor Kappa B; NO, Nitric Oxide; NOX, NADPH Oxidase; Nrf2, Nuclear Factor Erythroid 2-related Factor 2; OCP, Organochlorine Pesticide; p70S6K1, Ribosomal Protein S6 Kinase beta-1; PARP, Poly (ADP-ribose) Polymerase; PDS, Patient-Derived Spheroids; PI, Propidium Iodide; PMA, Phorbol 12-myristate 13-acetate; PRDX6, Peroxiredoxin 6; qPCR/qRT-PCR, Quantitative Reverse Transcription Polymerase Chain Reaction; Rac1, Ras-related C3 Botulinum Toxin Substrate 1; ROS, Reactive Oxygen Species; SCD1, Stearoyl-CoA Desaturase-1; shRNA, Short Hairpin RNA; siRNA, Small Interfering RNA; SOC, Serous Ovarian Cancer; SOD, Superoxide Dismutase; SREBP, Sterol Regulatory Element-Binding Protein; STAT3, Signal Transducer and Activator of Transcription 3; TIMP, Tissue Inhibitor of Metalloproteinases; TNF-α, Tumor Necrosis Factor-alpha; Trx1/TXN, Thioredoxin; TUNEL, Terminal deoxynucleotidyl transferase dUTP Nick End Labeling; ULA, Ultra-Low Attachment; VEGF, Vascular Endothelial Growth Factor; WT, Wild Type; XTT, 2,3-Bis-(2-methoxy-4-nitro-5-sulfophenyl)-2H-tetrazolium-5-carboxanilide; β-HCH, β-Hexachlorocyclohexane.

### 3.1 Tumor initiation/carcinogenic priming

ROS may contribute to the “early priming” stage of carcinogenesis by inducing inflammatory cytokines and DNA damage in normal or non-malignant epithelial cells. In particular, exposure to environmental toxicants or the accumulation of oxidative stress in tissues has been associated with an increased risk of tumor development. Various studies have investigated this relationship.

To determine whether organochlorine pesticide exposure is associated with changes in intracellular ROS levels in human ovary surface epithelial (HOSE) cells, researchers treated HOSE cells with 20 μM of β-hexachlorocyclohexane (β-HCH), dichlorodiphenyldichloroethylene (DDE), or dieldrin for 3 days and measured intracellular ROS levels using the redox-sensitive fluorescent dye, 2,7-dichlorodihydrofluorescein diacetate (H2DCFDA). β-HCH, DDE, and dieldrin significantly increased ROS levels by 71.06%, 80.82% (p < 0.01), and 65.59% (p < 0.05), respectively, compared with the control treatment (DMSO). The positive control (50 mM H_2_O_2_) significantly increased ROS levels by 78.44% (p < 0.01) compared with the DMSO control. These findings suggest that persistent exposure of human ovarian surface epithelial cells to β-HCH, DDE, or dieldrin can induce ROS generation. The results further showed that these treatments can stimulate the secretion of various inflammatory cytokines, thereby triggering chronic inflammatory responses and creating a tumor-promoting microenvironment that may further exacerbate genomic instability through DNA damage (15).

Although the mortality rate of serous ovarian cancer (SOC) is relatively higher in African American/Black women than in White women, the underlying reasons for this disparity remain unclear. Oxidative stress-induced DNA damage has been implicated in ovarian cancer, but its role in between-patient differences in SOC aggressiveness has not been fully clarified. To close this gap, researchers measured the levels of ROS, malondialdehyde (MDA), reactive carbonyl groups, and antioxidants in precancerous (cystadenoma, borderline) and invasive (stage III/IV) ovarian tissue samples obtained from African American and Caucasian subgroups. The study included normal ovarian tissues as negative controls (n=9), benign cystadenoma (n=7), borderline tumors (n=7), and invasive carcinoma (n=11). ROS concentrations in malignant tissues were approximately 96% higher than those in normal controls. Furthermore, ROS levels in African American women were about 9% higher than those observed in Caucasian women. MDA levels increased exponentially from normal controls and precancerous tissues to malignant tissues. In addition, malignant serous ovarian tissue samples showed significantly higher reactive carbonyl contents compared with non-diseased controls (p = 0.009), while GPX3 levels were markedly decreased in serous cystadenoma and malignant tissue samples, as well as in non-diseased controls, compared with borderline tumors. These findings suggest that the accumulation of ROS and MDA may act as contributing factors in the development of SOC (16).

### 3.2 Tumor progression: proliferation, survival, angiogenesis, and metabolic reprogramming

**Figure 2:**
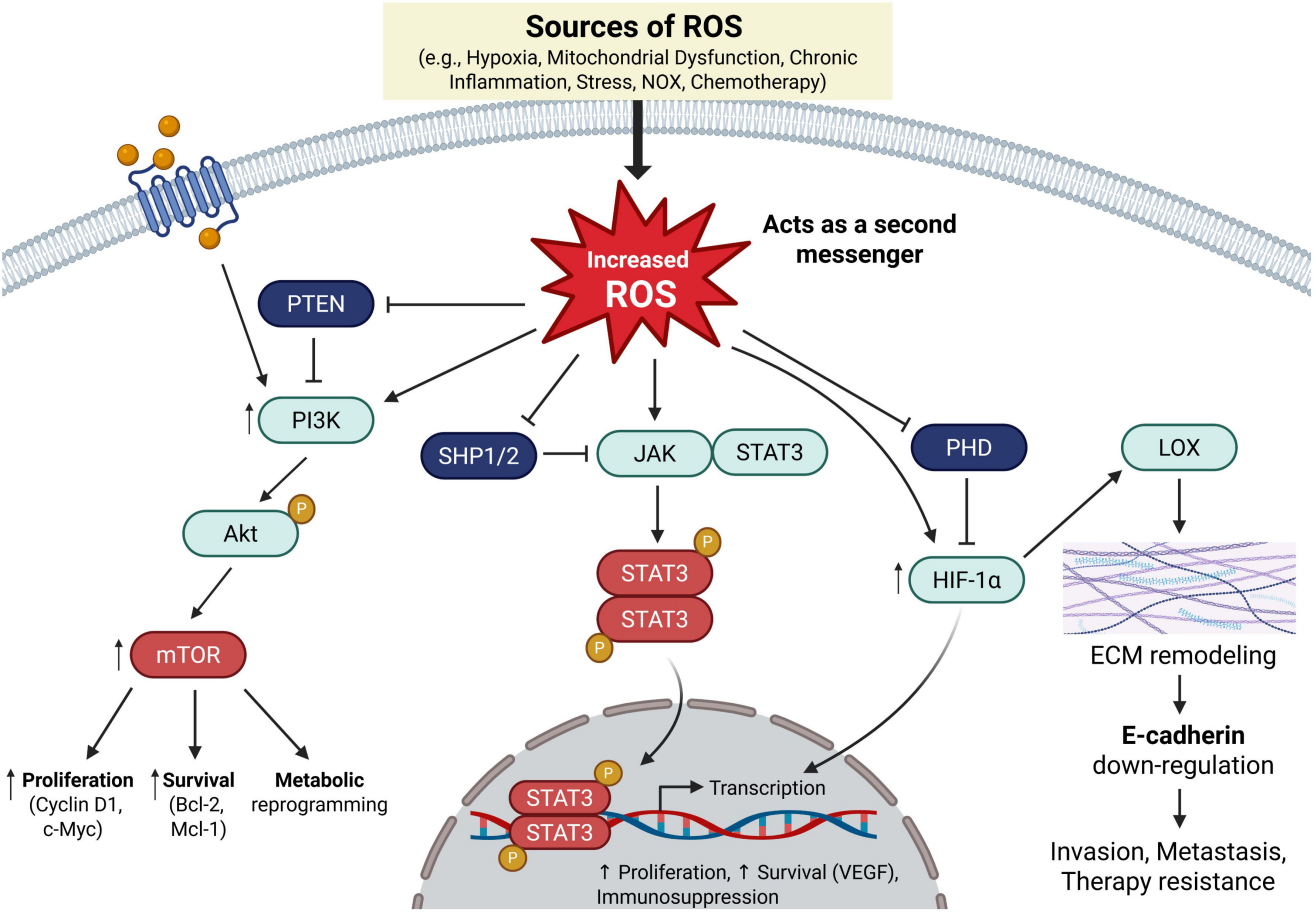
Mechanistic diagram: ROS-mediated pro-tumorigenic signaling in HGSOC.

During the tumor-progression stage, ROS function as downstream mediators of growth factor, hormonal, and hypoxic signaling. Through pathways such as those involving HIF-1α/VEGF, JAK/STAT3, and AKT/mTOR, ROS promote proliferation, survival, angiogenesis, and metabolic adaptation in tumor cells (**Figure 2**). In addition, redox buffering systems—including antioxidant enzymes and extracellular redox regulation—as well as modulation of the immune microenvironment may sustain tumor progression. Compared with normal or non-malignant epithelial cells, ovarian cancer cells may exhibit a basal redox state that is already shifted toward oxidative stress. An *in vitro* analysis comparing HOSE 642 cells and NEEC (normal epithelial endometrial cells) with two ovarian cancer cell lines (OVCA420 and OVCA429) and two endometrial cancer cell lines (ECC-1 and Ishikawa), revealed that cancer cells exhibit higher basal intracellular ROS production. In addition, markers of oxidative damage—including protein carbonyls, nitrotyrosine, and lipid peroxidation (MDA)—were also elevated in the cancer cells. These findings suggest that redox imbalance accompanied by ROS accumulation and oxidative damage may exist as a background condition during the progression stage of gynecologic cancers, including HGSOC. Such a redox environment may provide a basis for ROS-dependent signaling pathways to subsequently drive progressive phenotypes such as proliferation, survival, and angiogenesis (17, 18).

Within the tumor microenvironment, hormonal signaling serves as a critical upstream trigger that drives HGSOC progression through ROS-dependent pathways. HGSOC is widely considered to originate from the fallopian tube epithelium, where continuous exposure to follicular fluid—containing high levels of sex hormones and ROS during ovulation—induces genomic instability. In this context, both estrogen and androgens (such as testosterone) are highly relevant to redox dysregulation. Nine types of estrogen are present in human blood; of these, the three major forms are 17β-estradiol (E2), estrone (E1), and estriol (E3). E2 is the most abundant circulating estrogen and the most biologically active ovarian steroid (19, 20). It is also the most potent estrogen in terms of its effects on cell proliferation, apoptosis, and metastasis (21, 22). Furthermore, HGSOC cells express 17β-hydroxysteroid dehydrogenase (17β-HSD), which can convert the weak androgen, androstenedione, into testosterone (23). Estrogen receptors have been identified in mitochondria, and estrogen, alongside elevated testosterone, synergistically influences mitochondrial biogenesis and metabolism; thus, these hormones actively induce ROS production and exacerbate the oxidative stress phenotype (19, 24, 25). Finally, oxidative DNA damage and ROS generated by 17β-estradiol can induce lipid peroxidation and the oxygen radical-mediated oxidation of amino acid residues in proteins, resulting in the formation of carbonyl-containing functional groups (26, 27).

E2 exerts particularly strong effects on cell proliferation, apoptosis, and metastasis. To investigate the effects of E2 on the production of ROS and nitric oxide (NO) in HGSOC cells, the ovarian adenocarcinoma cell line OVCAR-3 was cultured and treated with various concentrations of E2, antioxidants (N-acetyl-L-cysteine [NAC] and ebselen), and the estrogen receptor antagonist, ICI182780. The results showed that intracellular peroxide levels increased in E2-treated cells, with the highest ROS production observed at a concentration of 100 nM E2. The addition of the antioxidants, NAC and ebselen, suppressed E2-induced ROS generation. The estrogen receptor antagonist, ICI182780, significantly inhibited E2-induced ROS production. When OVCAR-3 cells were treated with 100 nM E2 in combination with progesterone, ROS generation was significantly reduced compared with the E2 monotherapy group (p < 0.001). E2 treatment also increased NO production in OVCAR-3 cells, with 1000 nM identified as the most effective concentration (p < 0.001). The increase in ROS production was accompanied by enhanced cell viability, suggesting that estrogen-induced ROS may contribute to HGSOC progression (28).

An *in vitro* study using HGSOC cell lines (A2780 and SKOV-3) and non-malignant cell lines (MIHA and IOSE-80) evaluated ROS levels and related signaling and functional changes using H2DCFDA assays, Western blotting, siRNA knockdown, MTT assays, JC-1 staining, and flow cytometry. The results showed that HIF-1α significantly increased intracellular ROS levels, primarily that of hydrogen peroxide (H_2_O_2_; p < 0.01), and activated the JAK/STAT3 signaling pathway. The results demonstrated that ROS mediate HIF-1α-induced proliferation, invasion, and anti-apoptotic effects in HGSOC cells. Treatment with the antioxidant, NAC, reduced ROS levels and reversed these phenotypes. These findings provide *in vitro* evidence of a causal link through which the HIF-1α/ROS (H_2_O_2_)/JAK/STAT3 axis promotes HGSOC progression (29).

Follicle-stimulating hormone (FSH) and its receptor have been implicated in tumor angiogenesis and are considered risk factors for ovarian epithelial cancer (OEC). FSH has been reported to induce the expression of vascular endothelial growth factor (VEGF) and HIF-1α. The authors initially demonstrated that FSH induces the generation of ROS and activates Nrf2 signaling. They then examined whether FSH induces VEGF expression through a ROS-mediated Nrf2 signaling pathway. FSH significantly increased intracellular ROS levels (p < 0.01), which was accompanied by activation of Nrf2 and HIF-1α signaling and increased VEGF expression. These findings suggest that abnormal expression of ROS and Nrf2 plays an important role in FSH-induced angiogenesis in ovarian epithelial cells. The authors further proposed that ROS scavenging or Nrf2 inhibition may represent potential therapeutic targets for treating HGSOC (30).

The epidermal growth factor (EGF) and EGF receptor (EGFR) family members are frequently overexpressed in various human cancers, including HGSOC. Although ROS are known to participate in intracellular signaling processes, their role in EGF-induced angiogenesis and carcinogenesis remains incompletely understood. To close this gap, researchers investigated the role of ROS in regulating AKT, p70S6K1, VEGF, and HIF-1 in HGSOC cells. OVCAR-3 cells were treated with EGF and the H_2_O_2_ scavenger, catalase. EGF treatment increased H_2_O_2_ production, which activated the AKT/p70S6K1 pathway to transcriptionally enhance VEGF expression. Inhibition of H_2_O_2_ production by catalase suppressed the EGF-induced activations of AKT and p70S6K1, as well as the HIF-1-mediated expression of VEGF. Collectively, the results of this study demonstrated that (1) H_2_O_2_ generation is essential for the EGF-induced activation of the PI3K/AKT/p70S6K1 pathway (2), H_2_O_2_ transcriptionally mediates EGF-induced VEGF expression through AKT/p70S6K1 signaling, and (3) both p70S6K1 activation and H_2_O_2_ production are essential for ovarian tumor angiogenesis (31).

In another study examining ROS regulation in HGSOC cells, ROS levels were measured using H_2_O_2_-based fluorescent detection methods, inhibition of NOX-related activity (e.g., via DPI and rotenone) was performed, and HIF-1α and VEGF expression levels were analyzed by Western blotting and qPCR. The results showed that expression of the NADPH oxidase isoform, NOX4, was markedly higher in HGSOC cells. Specific inhibition of the NADPH oxidase subunit, p47phox, significantly reduced ROS production. ROS were found to regulate the expression levels of HIF-1 and VEGF in HGSOC cells. Increased endogenous ROS levels were required for the induction of angiogenesis and tumor growth. Furthermore, inhibition of NOX4 in HGSOC cells led to decreases in VEGF levels, HIF-1α levels, and tumor angiogenesis. Based on these findings, the authors proposed a novel mechanism underlying increased ROS production in HGSOC cells and demonstrated that endogenous ROS play important roles in promoting angiogenesis and tumor growth (32).

Lysophosphatidic acid (LPA) is produced by tumor cells and is present in the ascitic fluid of HGSOC patients. To investigate the role of ROS and NOX-related activity under LPA stimulation, researchers analyzed ROS production and functional indicators associated with proliferation and migration in HGSOC. In the HGSOC cell line, SKOV-3, treatment with the LPA receptor antagonist, VPC32183, suppressed cell growth and induced apoptosis. Exogenous LPA further enhanced ERK phosphorylation, Akt phosphorylation, and NF-κB activation. To determine whether ROS (known as secondary messengers in cellular signaling) participate in LPA signaling, cells were treated with the NADPH oxidase inhibitor, diphenyleneiodonium (DPI), and antioxidants such as NAC, EUK-134, and curcumin. All of these treatments inhibited LPA-dependent NF-κB activation and cell proliferation. LPA stimulated dichlorofluorescein fluorescence except in the presence of DPI, apocynin (a specific inhibitor of NADPH oxidase), VPC32183, or PEG-catalase. The results of this study suggest that NADPH oxidase is a major source of ROS and H_2_O_2_ plays an important role in LPA-mediated signaling pathways (33).

MIEF2 (mitochondrial elongation factor 2) is a key regulator of mitochondrial fission. Bioinformatics analysis showed that high expression of MIEF2 predicts poor prognosis in patients with HGSOC. However, the relationship between MIEF2 and abnormal lipid metabolism in HGSOC has not yet been fully clarified. To close this gap, researchers analyzed lipid metabolism reprogramming in HGSOC cells centered on MIEF2 by examining ROS production, AKT/mTOR pathway activation (p-AKT and p-mTOR), lipid synthesis-related proteins (SREBP1/2, ACC1, FASN, and SCD1), and functional outcomes such as cell proliferation and migration. The results indicated that increased mitochondrial ROS production and the consequent activation of the AKT/mTOR signaling pathway contributed to upregulating SREBP1 and SREBP2 in HGSOC cells. Functional analyses further demonstrated that MIEF2-regulated fatty acid synthesis and cholesterol biosynthesis play important roles in HGSOC progression. Taken together, the findings of this study suggest that MIEF2 promotes HGSOC growth and metastasis by activating ROS/AKT/mTOR signaling, increasing SREBP1 and SREBP2 expression, and enhancing fatty acid and cholesterol biosynthesis (34).

Superoxide dismutase (SOD) is an important antioxidant enzyme responsible for the removal of superoxide radicals (O_2_^-^). Manganese-containing SOD (Mn-SOD) is known to have tumor-suppressive functions and is localized in the mitochondria, where most superoxide radicals are generated during cellular respiration. In a study investigating how alterations in Mn-SOD (SOD2) affect the mitochondrial ROS balance (e.g., O_2_^-^) and tumor cell adaptation, ROS levels and survival/adaptation-related indicators (ROS assays, protein analyses, and cell viability tests) were evaluated in HGSOC cells following modulation of Mn-SOD expression. The results showed that Mn-SOD protein expression levels were significantly higher in malignant tissues compared with normal tissues (p < 0.05). Suppression of Mn-SOD expression using small interfering RNA increased superoxide production by approximately 70% in HGSOC cells, which led to enhanced cell proliferation *in vitro* and increased aggressiveness of tumor growth *in vivo*. Furthermore, stimulation of mitochondrial O_2_^-^ generation induced increased Mn-SOD expression. These findings indicate that increased Mn-SOD expression in HGSOC represents a cellular response to intracellular ROS stress, and that SOD-mediated superoxide removal can alleviate ROS stress and reduce the ability of ROS to stimulate cell growth (35).

Glutathione peroxidase 3 (GPx3) is an extracellular glutathione peroxidase known to play dual roles in cancer. In a study examining the role of GPx3, researchers manipulated extracellular redox conditions in HGSOC cells using factors such as extracellular H_2_O_2_ and ascorbate, and evaluated cellular survival/adaptation-related functional indicators. High GPx3 expression was associated with reduced overall patient survival and increased tumor stage. Suppression of GPx3 expression reduced clonogenic capacity and anchorage-independent cell survival. In addition, inhibition of GPx3 expression significantly increased the amount of H_2_O_2_ in the culture media of cells treated with 0.3 mM oxidant, indicating that GPx3 expression is important for removing excess extracellular H_2_O_2_. These findings suggest that GPx3 protects HGSOC cells from external oxidative damage by scavenging H_2_O_2_. Overall, the study demonstrated that high-grade serous carcinoma (HGSC) ovarian cancers can be clearly divided into groups according to GPx3 expression levels, and that GPx3 is essential for the survival of HGSC ovarian cancer cells in the unique ascitic tumor microenvironment and protect cells from extracellular oxidative stress factors (36).

Galectin-3, a β-galactoside-binding lectin, is involved in various immune processes and has been associated with poor prognosis in several types of cancer. To investigate the role of galectin-3 in the HGSOC tumor microenvironment, researchers examined its effects on the interaction between natural killer (NK) cells and neutrophils. Ascitic fluid from the metastatic tumor microenvironment and cystic fluid from primary tumor sites were collected from patients with HGSC, along with peripheral blood samples. Galectin-3 concentrations were measured in ascites, cystic fluid, serum, and/or plasma. In conditions with high galectin-3 levels, ROS production, antioxidant pathways (including those involving SOD1 and thioredoxin), and immune-related responses were analyzed. The results showed that galectin-3-induced ROS generation in neutrophils reduced NK cell viability and weakened antitumor immune responses. These findings suggest that the elevated levels of galectin-3 detected in the HGSC microenvironment may impair the tumor cell-killing ability of NK cells through a ROS-dependent mechanism (37).

### 3.3 Metastatic competence

Metastatic competence differs from simple proliferation and refers to the acquisition of invasive and migratory capabilities, including epithelial-mesenchymal transition (EMT) marker alterations, extracellular matrix (ECM) remodeling (e.g., lysyl oxidase, LOX), and matrix metalloproteinase (MMP) production. *In vitro* studies suggest that ROS can enhance metastasis-related capabilities by regulating HIF-1α-mediated EMT-like changes and MMP expression. To investigate this in SKOV-3 ovarian cancer cells, ROS levels were measured using DCFH-DA, the expression levels of HIF-1α, LOX, and E-cadherin were analyzed using Western blotting and qPCR, and cell migration and invasion were evaluated using appropriate assays. After exposure of SKOV-3 cells to emodin, intracellular ROS levels increased by 55.7% compared with those in untreated controls (p < 0.01). In contrast, treatment with the known ROS scavenger, dithiothreitol (DTT), significantly reduced ROS levels by 41.2% (p < 0.01). Emodin significantly increased the expression of HIF-1α and LOX, whereas co-treatment with DTT suppressed these inductions. Conversely, E-cadherin expression was markedly suppressed in emodin-treated cells but was restored when DTT was co-administered. These findings suggest that ROS may promote the acquisition of EMT-associated metastatic potential through the HIF-1α/LOX/E-cadherin signaling axis (38).

MMPs are calcium-dependent, zinc-containing endopeptidases that play important roles in tumor cell migration and invasion. The Rac1 protein can influence cell migration and invasion primarily through the generation of ROS. Therefore, targeting MMP activity and regulating the Rac1/ROS/MAPK/AP-1 signaling pathway may represent a novel therapeutic strategy. Rhein inhibits the migration and invasion of SKOV-3 PM4 cells by scavenging intracellular ROS. Researchers applied rhein to these cells and analyzed Rac1 activation, ROS levels, MAPK/AP-1 signaling, and the productions of MMP-2, -3, -7, -9, and -19. The Rac1 activator, phorbol 12-myristate 13-acetate (PMA), significantly increased the protein expression levels of MMP-2, -3, -9, and -19, whereas rhein and the Rac1 inhibitor, NSC23766, produced opposite effects. Rhein was also found to reduce ROS production and decrease NADPH oxidase activity. Furthermore, rhein significantly suppressed the phosphorylation of JNK and AP-1 in PMA-treated cells. Collectively, these findings indicate that rhein acts as a potential inhibitor of Rac1 and regulates MMPs and proteins associated with Rac1/ROS/MAPK/AP-1 signaling (39). While these findings suggest that ROS act as upstream regulators coordinating metastasis-related effectors, it is important to note that these specific *in vitro* observations are currently derived exclusively from SKOV-3 models. Given that recent genomic profiling indicates SKOV-3 may not strictly represent HGSOC, further investigations using diverse and clinically representative HGSOC cell lines are required to fully validate these ROS-driven metastatic mechanisms.

### 3.4 Therapeutic response

Regulation of molecular mechanisms under hypoxic conditions plays an important role in HGSOC progression. Mitochondria continuously undergo cycles of fission and fusion to enable cells to survive while adapting to environmental changes. Dysregulation of mitochondrial dynamics has been reported in various diseases, including cancer. A study investigated the relationship between mitochondrial dynamics and sensitivity to cisplatin (CDDP) in HGSOC cells under hypoxic conditions (<1% O_2_). The results showed that hypoxia promoted mitochondrial fission and increased resistance to CDDP in ovarian cancer cells. Hypoxia-induced ROS increased mitochondrial fission, whereas NAC- or Trolox-mediated free-radical scavenging suppressed this response. In addition, H_2_O_2_ treatment reduced the inhibitory phosphorylation of Drp1 at Ser637 and increased mitochondrial fission. Inhibition of mitochondrial fission enhanced the sensitivity of hypoxic ovarian cancer cells to CDDP. These findings indicate that hypoxia-induced ROS promote mitochondrial fission by regulating Mfn1 protein expression and Drp1 activation (40).

The acquisition of drug resistance is a major obstacle in the clinical use of platinum-based chemotherapy for treating HGSOC. Using quantitative high-throughput combination screening (qHTCS) and RNA-sequencing (RNA-seq), researchers demonstrated that dual oxidase maturation factor 1 (DUOXA1) is overexpressed in platinum-resistant HGSOC cells, leading to excessive production of ROS. Elevated ROS levels sustained activation of the ATR-Chk1 pathway, which in turn contributed to cisplatin resistance in ovarian cancer cells (41).

Using a Seahorse XF24 extracellular flux analyzer, researchers measured the oxygen consumption rate (OCR) and extracellular acidification rate (ECAR), and revealed that cisplatin-resistant ovarian cancer cells exhibited OCR levels approximately three to four times higher than those of parental ovarian cancer cells. This finding suggested that cisplatin-resistant cells have undergone a dramatic metabolic shift toward oxidative phosphorylation. In addition, treatment with the mitochondria-targeted antioxidant, mito-Tempo, significantly reduced cellular ROS levels. The results supported the idea that increased mitochondria-derived ROS may be responsible for the elevated activation of calcium/calmodulin-dependent protein kinase II gamma (CAMK2G) in cisplatin-resistant cells. Furthermore, short-term pretreatment with NAC significantly increased the sensitivity of cisplatin-resistant cells to cisplatin treatment and reduced the cisplatin-sensitizing effect of CAMK2G inhibition. These findings suggest that endogenous ROS act upstream of CAMK2G and contribute to intrinsic cisplatin resistance in ovarian cancer cells (42).

To examine whether the loss of mitogen-activated protein kinase phosphatase 3 (MKP3) under an oxidative stress condition enhances tumorigenicity and chemoresistance, researchers evaluated MKP3 expression, ubiquitin-proteasome-related changes, and cisplatin response in ovarian cancer cells. Knockdown of endogenous MKP3 using short hairpin RNA (shRNA) increased ERK1/2 activation, cell proliferation, anchorage-independent growth, and cisplatin resistance in ovarian cancer cells. In contrast, forced expression of MKP3 in MKP3-deficient ovarian cancer cells markedly reduced ERK1/2 activation and suppressed cell proliferation, anchorage-independent growth, and tumor formation in nude mice. Moreover, overexpression of MKP3 increased the sensitivity of ovarian cancer cells to cisplatin-induced apoptosis both *in vitro* and *in vivo*. These findings suggest that there is a molecular mechanism by which ROS accumulation during HGSOC progression induces MKP3 degradation, leading to abnormal ERK1/2 activation and contributing to tumorigenicity and chemoresistance in human HGSOC cells (43).

Collectively, these distinct mechanisms do not operate in isolation but rather form a highly integrated, adaptive network that drives ROS-mediated therapy resistance in HGSOC. In this conceptual model, chemotherapy (e.g., cisplatin) initially induces an acute ROS surge, triggering immediate DNA damage responses such as ATR-Chk1 signaling to stall the cell cycle and prevent apoptosis. Concurrently, to survive this continuous oxidative stress, cancer cells activate kinases like CAMK2G and undergo metabolic reprogramming via mitochondrial fission. This structural mitochondrial adaptation efficiently distributes the redox burden and prevents fatal ROS accumulation. From a clinical perspective, this mitochondrial redox-buffering capacity is likely the most significant determinant of chemoresistance. Therefore, combinatorial therapeutic strategies designed to dismantle these adaptive mitochondrial dynamics alongside standard platinum-based therapy hold the greatest clinical promise for resensitizing resistant HGSOC tumors.

In a translational context, while general non-specific antioxidants (e.g., conventional vitamins) have largely failed in clinical trials due to their inability to reach effective intracellular concentrations and their potential to inadvertently protect cancer cells, next-generation candidates show distinct promise. Specifically, mitochondria-targeted antioxidants, such as Mito-TEMPO and MitoQ, selectively accumulate in the mitochondrial matrix to precisely dismantle the redox-buffering adaptations of chemoresistant HGSOC cells. Furthermore, the strategic application of ROS-scavengers like N-acetylcysteine (NAC) in targeted delivery systems provides a translational framework for resensitizing tumors to platinum-based therapies without compromising systemic redox homeostasis.

### 3.5 Limitations and future perspectives

Although the current review provides comprehensive insights into ROS-mediated pro-tumorigenic mechanisms, several limitations should be noted. First, the relatively small number of included studies (n=19) is a direct consequence of our strict inclusion criteria aimed at isolating pro-tumorigenic mechanisms; thus, these findings should be interpreted as a focused mechanistic synthesis rather than an exhaustive systematic review. Second, many of the included studies relied on DCF-based probes that measure “total oxidative signals,” making it difficult to distinguish specific ROS species and their sources. Third, widely used antioxidants such as NAC may exert nonspecific effects, and variations in cell lines and culture conditions can influence redox homeostasis. Importantly, while recent genomic profiling indicates that some historically broad ovarian cancer cell lines (e.g., SKOV-3, A2780) may not strictly represent HGSOC, early foundational *in vitro* studies—particularly those evaluating metastatic competence—heavily relied on these models. Therefore, to ensure academic transparency, we have explicitly specified the cell line subtypes in **Table 1**. Future research should seek to standardize experimental systems within consistent, clinically representative HGSOC models to more precisely clarify when and where ROS-dependent causal circuits operate. In addition, functional endpoints—such as proliferation, EMT/invasion, angiogenesis-related factors, and drug responses—should be systematically integrated to clarify how ROS-dependent causal circuits critically operate at each stage of HGSOC development.

## 4 Conclusions

**Figure 3:**
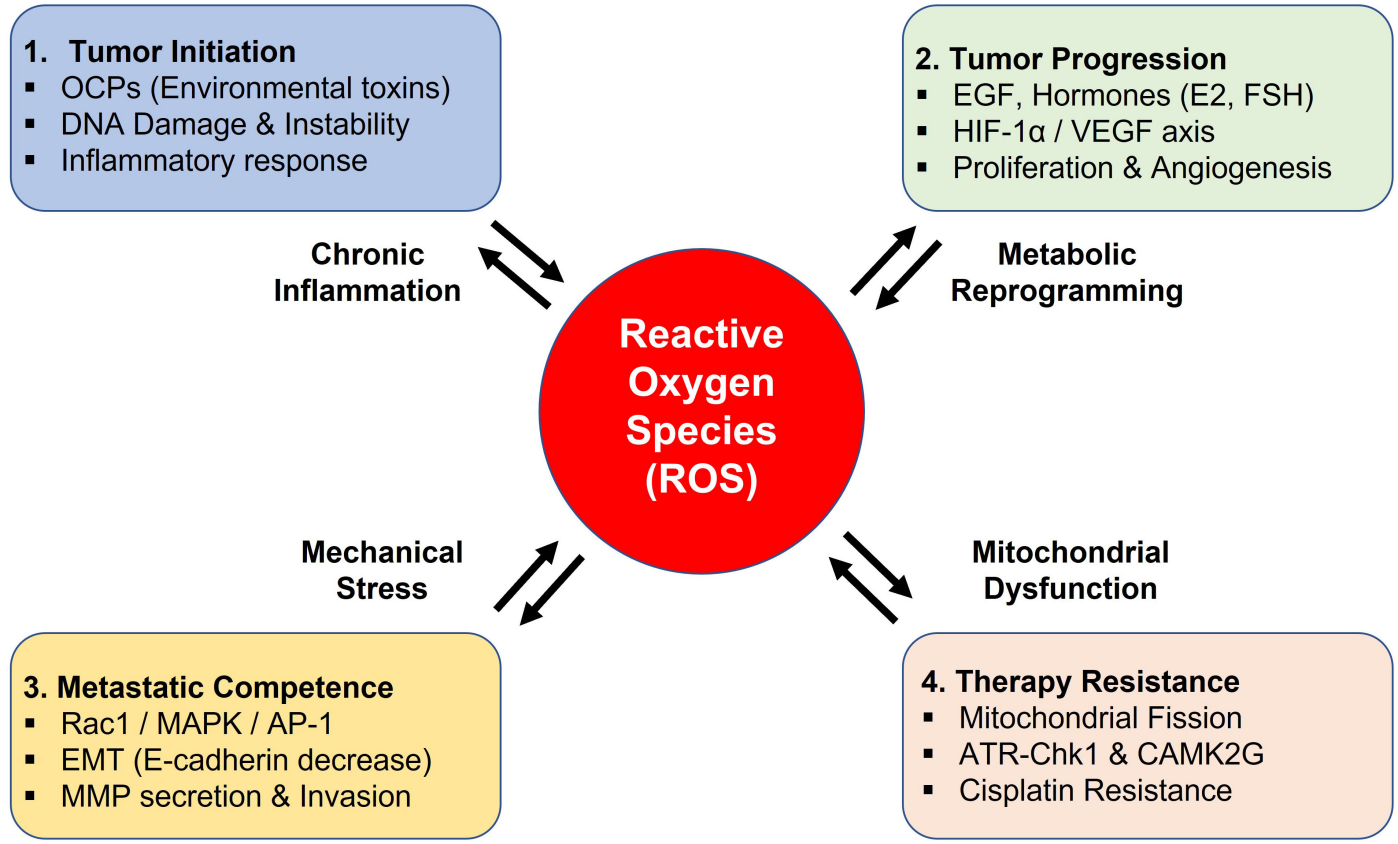
The critical role of ROS across all stages of HGSOC pathophysiology.

Taken together, the *in vitro* evidence reviewed herein indicates that ROS are repeatedly observed to activate signaling circuits supporting HGSOC cell proliferation, survival, angiogenesis, invasion/metastasis, and therapeutic resistance. Upstream stimuli—such as environmental toxicants, hormonal and growth factor stimulation, hypoxia, microenvironmental lipid signaling, and metabolic reprogramming—induce or amplify ROS. During the initiation and early stages of tumors, the studies show that increased ROS levels under organochlorine pesticide exposure are accompanied by induction of inflammatory cytokines and DNA damage markers, suggesting that a ROS-mediated inflammation-genomic damage axis may function as an early event that creates a carcinogenic “priming” environment in ovarian epithelial cells. During tumor progression, emerging evidence links ROS to survival and growth signaling pathways, such as those involving the HIF-1α/VEGF axis, JAK/STAT3, and AKT/mTOR, which can strengthen phenotypes related to cell proliferation, anti-apoptosis, and angiogenesis. In particular, physiologically relevant upstream stimuli, such as hypoxia or hormonal stimulation, induce HIF-1α and VEGF through ROS, and these phenotypes are attenuated by interventions such as NAC. This supports the concept that ROS function not merely as metabolic byproducts but also as functional intermediates in progression-related signaling pathways. With respect to metastatic competence, studies using *in vitro* models have shown that ROS are associated with EMT-related marker changes (e.g., decreased E-cadherin), extracellular matrix remodeling (LOX), and regulation of MMPs, which can enhance migration and invasion programs. These findings suggest that ROS may act as upstream regulators to coordinate metastasis-related effectors. In the context of therapeutic response and resistance, accumulating evidence indicates that ROS contribute to the maintenance and reinforcement of resistance to platinum-based chemotherapy through adaptive mechanisms such as activation of the DNA damage response (ATR-Chk1), remodeling of ROS-regulated phosphorylation networks (e.g., CAMK2G-ITPKB), alterations in mitochondrial dynamics, and loss of MAPK regulators such as MKP3. These observations imply that ROS function not simply as indicators of oxidative stress during treatment, but also may converge on specific signaling nodes—such as DNA damage response pathways, kinase networks, and mitochondrial remodeling—to stabilize drug-resistant cellular states (**Figure 3**).

Therefore, based on current *in vitro* evidence, ROS are likely to function as pathophysiological mediators that translate diverse upstream stimuli into a common progression program across multiple stages of HGSOC, including initiation, progression, metastasis, and therapeutic resistance. Ultimately, the accumulated knowledge regarding the diverse mechanisms and clinical manifestations associated with ROS expression in HGSOC will contribute to a more precise understanding of its pathophysiology. Furthermore, research on redox-targeted therapeutic strategies, particularly the application of mitochondria-targeted antioxidants, may provide valuable translational insights and contribute to the development of improved therapeutic approaches not only for HGSOC but also for other ROS-related diseases.
